# Vitamin D Deficiency as a Possible Cause of Type 1 Diabetes in Children and Adolescents up to 15 Years Old: A Systematic Review

**DOI:** 10.1900/RDS.2022.18.58

**Published:** 2022-06-30

**Authors:** Maria Daskalopoulou, Magdalini Pylli, Konstantinos Giannakou

**Affiliations:** 1 Department of Health Sciences, School of Sciences, European University Cyprus, Nicosia, Cyprus,; 2 Department of Health Sciences, School of Sciences, European University Cyprus, Nicosia, Cyprus. HIV Surveillance Department, Hellenic National Public Health Organization, Greece,; 3 Department of Health Sciences, School of Sciences, European University Cyprus, Nicosia, Cyprus.

**Keywords:** vitamin D, cholecalciferol, diabetes type 1, type 1 diabetes mellitus, children, adolescents, systematic review

## Abstract

**OBJECTIVE:**

To evaluate vitamin D ((25OH)D levels) deficiency as a possible cause in the development of DT1 in children and adolescents aged 0-15.

**METHODS:**

We searched PubMed/ Medline, EBSCO, and Cochrane Library to identify potentially eligible articles that examine whether low serum 25(OH)D levels are associated with subsequent development of DT1. All type of research designs, including randomized and non-randomized controlled trials, prospective and retrospective cohort studies, case-control and cross-sectional studies with subjects aged ≤15 years old were consider for inclusion.

**RESULTS:**

Seven original studies met the entry criteria. Most of these studies found up to 50% lower levels of vitamin D in children with DT1 compared to control group and a significant positive association between vitamin D levels and of the risk of developing DT1. Results of quality assessment demonstrated moderate to high quality of all the studies included.

**CONCLUSIONS:**

Vitamin D deficiency may be a possible cause in the development of DT1 in the early years of life and particularly in children with genetic predisposition, whilst the deficiency of vitamin D is a very common occurrence in patients with DT1. Further long-term studies on children are required to determine the role of vitamin D on DT1.

## 1 Introduction

Diabetes mellitus is an endocrine disorder that belongs to the group of diseases characterized by high blood glucose levels. Diabetes mellitus Type 1 (DT1) is an autoimmune condition developed due to a dysfunction of pancreatic beta-cells. They are initially disrupted and their eventual, full dysfunction in the long-term results in their inability to produce insulin and consequently the need for exogenous insulin administration [1,2]. The symptoms of diabetes mellitus (DM) include polyuria, polydipsia, fatigue, and weight loss. Most of the symptoms are not severe, but over time, they can cause damage or even complete destruction of an organ, resulting in irreversible conditions such as blindness, amputation, stroke, heart attack, kidney failure, and death [3,4]. Computational techniques used for diabetes show that DT1 is also associated with increased risk of fracture in human bone [5] and foot ulceration [6,7]. More than 90% of newly diagnosed people with DT1 have measurable antibodies that act against specific beta-cells proteins, such as insulin. In studies focused on newborns at birth, the first autoantibody appearance was observed before the second year of life [1]. People with DT1 represent 5%-10% of all patients with DM [8]. The US Centers for Disease Control and Prevention (CDC) epidemiological report states that in 2018, 210,000 children and adolescents under 20 years of age in the US (25 persons/10,000) were diagnosed with DM, of which 187,000 were diagnosed with DT1 [9].

DT1 is a multifactorial disease, in which various environmental factors (viral infections, premature infancy versus breastfeeding, toxins) [10] interact with genetic predisposition to trigger an autoimmune reaction against beta-cells [11]. Vitamin D deficiency is a powerful nutritional factor contributing to beta-cell autoimmunity and the development of DT1, especially in the early stages of life [12-14]. Vitamin D is a fat-soluble vitamin with two natural sources. The first is a nutritional source. Vitamin D is obtained in the form of D_3_ (cholecalciferol) from certain animal products, such as fatty fish, and in the form of D_2_ (ergocalciferol) contained in yeast and mushrooms. The second and main source of vitamin D is ultraviolet sunlight (UVB), which provides the human body the vitamin in the form D_3_ [15,16].

There is increasing evidence showing that vitamin D deficiency contributes to the pathogenesis of DT1, indicating that hypovitaminosis D is an important environmental factor for the development of DT1 [17]. Vitamin D receptors are found on the surface of almost all human cells, a distribution that allows its multilateral action. Several mechanisms mediate, which complicate the pathophysiology of DT1, leading to the destruction of beta-cells. These mechanisms include the presence of CD8 + T lymphocytes, macrophages, neutrophils, and natural killer cells [18]. Calcitriol, which is the biologically active form of vitamin D, inhibits the differentiation and maturation of these dendritic cells, while promoting their apoptosis so that they cannot act as mature antigenic precenting cells (APCs), thereby preventing the destruction of beta-cells [19].

Vitamin D deficiency should be diagnosed directly and treated appropriately in the early years of life, especially in children with a family history of DT1 [16]. Considering the specificities of the child and adolescent population, as well as the lack of systematic reviews and meta-analyses for this age group, the aim of this systematic review was to evaluate the role of vitamin D levels in the development of DT1 in children and adolescents aged 0-15.

## 2 Methods

### 2.1 Search strategy and eligibility criteria

To conduct this systematic review, we followed the Preferred Reporting Items for Systematic Reviews and Meta-Analyses guidelines (PRISMA) [20]. Two researchers (MD & MP) independently searched PubMed/Medline, EBSCO, and Cochrane Library databases from inception through December 2020 to identify potentially eligible articles that examine the role of vitamin D levels in the development of DT1 by the age of 15 years. Discrepancies were resolved by mediation and discussion with a third author (KG). The following search terms were used individually or in various combinations: “vitamin D,” “cholecalciferol,” “diabetes type 1,” and “type 1 diabetes mellitus.” Additional information concerning the search strategy is presented in Supplementary Table 1. First, each article’s title and abstract identified through the search were examined, and then, the full texts of potentially eligible articles were reviewed for evaluation. A reference list of relevant studies was screened to identify additional studies. Unpublished reports and abstracts were not considered. Any disagreements about inclusion were resolved through consultation with a third reviewer (KG).

**Table 1.** Characteristics of studies included in the systematic review

Author, year	Study design	Country	Follow up period	Participants (boys/ girls)	Age of participants (years)	25(OH)D (nmol/l)	Association between 25(OH)D levels and T1D
**Norris et al. (2018)**	Casecontrol study	Colorado, Georgia/ Florida, Washington, Finland, Germany, Sweden	Birth period: 9/2004 - 2/2010 Follow up for DT1 development up to 31/5/2012	Childhood: cases: 376 (209/167) controls: 1041 Infancy: cases: 360 controls:360	Enrollment in the follow up before age 4 months. Enrollment in the follow up in age ≤12 months.	cases: 52,85 ± 17,18 controls: 58,16 ± 16,3 cases: 48,18 ± 21,9 controls: 52,11 ± 21,25	Childhood: Children with normal 25(OH)D in childhood have 32% lower odds [OR 0,68 (0,52-0,89)] of expressing autoantibodies and thus developing DT1 compared to children with 25(OH)D insufficiency (<50nmol/lt). Infancy: Children with normal 25(OH)D in infancy have 40% lower odds [OR 0,59 (0,44-0,79)] of expressing autoantibodies and thus developing DT1 compared to children with 25(OH)D deficiency (<50 nmol/lt).
**Rasoul et al. (2016)**	Casecontrol study	Kuwait	25(OH)D serum levels measurement in newly diagnosed DT1 patients, placed on a daily dose of insulin before the 15th birthday. No reference on date or time	cases: 215 (104/112) newly diagnosed T1D patients controls: 204 (106/98) healthy volunteers	cases: 9,58 ± 3,2 controls: 9,59 ± 2,7	cases: 34,6 ± 16,65 controls: 37,45 ± 26	99% of DT1 children had 25(OH)D insufficiency or deficiency. DT1 children have 4 times more odds to develop 25(OH) D insufficiency or deficiency [OR 4,176 (1,147-15,202) p=0,027] compared to healthy children.
**Jacobsen et al. (2016)**	a) casecohort study b) casecontrol study	Denmark	a) Birth period: 1981-2002, Follow up 1981- 2012. b) Birth period:1981-1999, DT1 onset up to 2004,follow up 1981-2004	a) cases: 912 (49,5% /50,5%) controls: 2873 (51,6% / 48,4%) b) cases: 527 (50,3% / 49,7%) controls: 527(54,3% / 45,7%)	a) cases: 0-31 Age of DT1 onset 10,2 (7,5-12,5) controls: 0-31 Age of T1D onset 10,5 (5,1-11,5) b) cases: 0-31 Age of DT1 onset 8,5 (5-11,4) controls:0-31	a) cases: 26,9 (17,8- 41,3) controls: 26,8 (16,5-36,7) b) cases: 24,8 (15,7- 35,4) controls: 21,1 (12-32,9)	a)25(OH)D levels are not associated to risk of developing DT1 up to 18 years old b) 25(OH)D at birth are not associated to risk of developing DT1 up to 18 years old [OR 0,86 (0,60-1,22)]
**Taalat et al. (2016)**	Casecontrol study	Saudi Arabia	6/2013-6/2015	cases: 250 controls: 250	cases: 8,5 ± 0,5 controls: 8,5 ± 0,5	cases: 30,92 ± 7,65 controls: 60,37 ± 25	Healthy children had 50% higher 25(OH)D [OR 0,51 (0,33- 0,68), p=0,001] compared to DT1 children. DT1 girls had significantly lower 25(OH)D [OR 0,50 (0,30-0,83)], p=0,008] compared to boys.
**Cadario et al. (2015)**	Casecontrol study	Italy	Birth period:1992-2012 Follow up period 2002- 2012 to assess the risk of DT1 development 0-10 years of age(< 10 years old)	cases: 67 controls: 236 *48% of total participants were boys	cases: 7 ± 2,5 (DT1 diagnosis age: 4,2 ± 2,5) controls: 7,2 ± 0,49	cases: <5,35 = 36 infants ≥5,35 = 31infants controls: <5,35 = 103 infants ≥5,35 = 133 infants	25(OH)D at birth is not associated to risk of developing DT1 up to 10 years old [OR 1,76 (0,92-3,38)]. 26% of the participants with DT1 were immigrants from North African countries. In this group of migrants, it was found that children 25(OH)D <5,35nmol/lt have 14 times more odds to develop DT1 up to 10 years old [OR 14,02 (1,76-111,7), p = 0,04] compared to children of the same group with 25(OH)D >5,35nmol/lt.
**Franchi et al. (2014)**	Casecontrol study	Italy	5/2010-7/2012 Newly diagnosed DT1 patients	cases: 58 (32/26) controls: 166 (92/74)	cases: 9,2 (1,1-16) controls: 8,7 (1,1-16,3)	cases: 36,2 (7,5- 121,0) controls: 48,7 (7,5-190,2)	Children with 25(OH)D 51-74nmol/lt have almost 4 times more odds to develop DT1 [OR 3,45 (1,02-11,72), p=0,010]. Children with 25(OH)D ≤50nmol/lt have nearly 6 times more odds to develop DT1 [OR 5,56 (1,1-16,3), p=0,010].
**Bener et al. (2009)**	Casecontrol study	Qatar	6/8/2007-25/12/2007	cases: 170 (88/82) controls: 170 (80/90)	cases: 10,5 ± 3,8 controls: 9,9 ± 3,8	cases: 39,5 ± 23 controls: 46,2 ± 24	Children with 25(OH)D ≤30nmol have 1.4 more odds to develop DT1 [OR 1,385 (1,0-1,91), p=0,048] compared to children with normal 25(OH)D.

All types of research articles, including randomized and non-randomized controlled trials, prospective and retrospective cohort studies, case-control studies, and cross-sectional studies with participants aged ≤15 years old, written in Greek, English, Italian, or Spanish, were eligible for inclusion. Studies used risk ratio (RR), odds ratio (OR), or hazard ratio (HR) as a measure of association between vitamin D levels and DT1 were considered for inclusion. No restriction on publication date or country of origin was applied. All studies that were evaluating the role of vitamin D in the development of DT1 assessing other factors than vitamin D levels were excluded. Animal studies also were excluded.

### 2.2 Data extraction and assessment of methodological quality

Two authors (MD & MP) extracted the data independently, and any discrepancy was resolved after consultation with a third author (KG). The extracted data included: first author, year of publication, country of origin, design of the study, characteristics of participants (sex and number of patients and individuals in the control group, age of the patients and controls, 25(OH)D levels of the patients and controls, and main results obtained by each study. We assessed the quality of the studies using the Newcastle-Ottawa Scale (NOS) [21], which assigns a maximum of nine points to each study – four points for selection, two points for comparability, and three points for exposure or outcome. Although the scale does not specify a certain cut-off score for low- or high-quality methodology, scores above 5 stars are deemed to have moderate-to-high quality. Discrepancies were resolved by mediation and discussion with a third author (KG).

### 2.3 Data synthesis

Because of substantial between-study heterogeneity among in terms of the exposure definitions, measurement methods, and follow-up period, no attempt was made at a formal meta-analysis. Therefore, we summarized the entire body of studies descriptively and performed a qualitative synthesis of them.

## 3 Results

**Figure 1.**
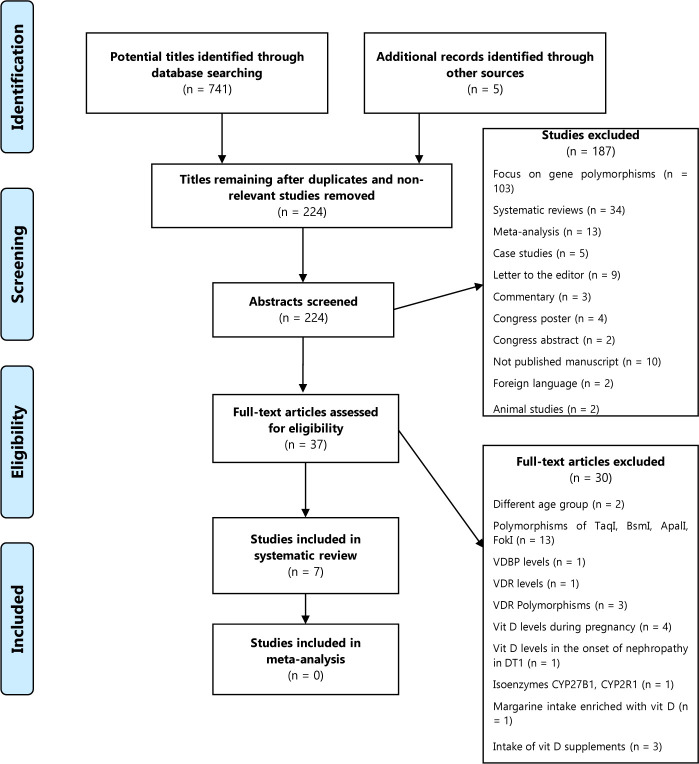
PRISMA flow diagram of the study selection process (Moher et al., 2009).

The flowchart in **Figure 1** shows the selection process of the articles included in the study. We identified a total of 741 references in the selected databases. We assessed 37 studies for inclusion based on the full text; ultimately, we determined that only 7 studies [22-28] were eligible for inclusion in this systematic review.

### 3.1 Demographic and clinical characteristics

**Table 1** presents the characteristics of the studies included in this systematic review. All studies were case-control studies, and one included a second part that was a case-cohort study. The maximum duration of participant follow-up was 31 years and the shortest was 6 months. The ratio of boys and girls was around 50% with small variations, except for one study [23], which refers only to the total number of participants, for both the cases group and the control group. The number of participants involved as cases was less than 100 in two studies [26,27], 100 to 500 participants in four studies [22-24,28], and more than 500 in one study [25]. The number of participants involved in control groups was not less than 100 persons in any study, 100 to 500 people in five studies [23,24,26-28], and more than 500 in two studies [22,25]. The sample of each study consisted of children and adolescents under 15 years of age. The age of DT1 development was not evaluated in all studies. One study reported that 20% of participants developed DT1 at the age of 4, 28% developed DT1 from 4-6 years old, and 52% at the age of 6 [24]. Two studies indicated that the age of appearance of DT1 among participants was 3.5±0.39 [23] and 4.2±3.4 years [26], respectively. All persons included in the group of cases had a DT1 with a certified medical diagnosis, whereas the criterion for persons included in the control group was not to have DT1. Yet, diagnostic criteria for the inclusion and exclusion of participants were different in each study and are presented in **Table 2**.

**Table 2.** Inclusion and exclusion criteria of participants of each study

STUDY	DIAGNOSTIC CRITERIA OF «CASES» GROUP	DIAGNOSTIC CRITERIA OF «CONTROLS» GROUP
**Norris et al. (2018)**	A) Infancy: • Infants <4 months old were screened for DT1 associated HLA genotypes. B) Childhood: • Developed persistent IA. Presence of at least one of the following autoantibodies: GADA, IAA, IA-2A	A) Infancy: • Infants from the general population, no first-degree relative with DT1 were eligible if they had any of the following HLADR- DQ genotypes: DR3-DQ2/DR4-DQ8, DR4-DQ8/DR4-DQ8, DR4-DQ8/DR8-DQ4, DR3-DQ2/DR3-DQ2. • If they had first-degree relative with DT1 were eligible if they had any of the above or one of the following HLA-DR-DQ genotypes: DR4-DQ8/DR4-DQ2.3, DR4-DQ8/DR1-DQB5.1, DR4-DQ8/DR13- DQ6.4, DR3-DQ2/DR9-DQ9.3, DR4-DQ8/DR9-DQ9.3. • “Case-control” matching was 1:1 for 8 «controls», 1:2 for 71 ‘‘controls’’and 1:3 for 297 ‘‘controls’’. B) Childhood: • Not developed persistent IA within ±45 days of the event time • Matching factors: clinical center, sex, family history of DT1 • “Case-control” matching was 1:3
**Rasoul et al. (2016)**	• DT1 diagnosis by physician • Placed on daily insulin dose before the 15^th^ birthday and a Kuwaiti national resident in the area at the time of first insulin administration	• Random selection • Healthy volunteers • Kuwaiti Nationals • Matched for ethnicity, gender, and age
**Jacobsen et al. (2016)**	A) Case-Cohort study: • Born 1981-2002 • DT1 diagnosis up to 2012 • Selected data and biobank material from Danish Childhood Diabetes Registry (DNSB) B) Case-Control study: • Born 1981-2002 • DT1 diagnosis from 1/1/1981 up to 1/5/2004 • Had a DBS card present in the DNSB Excluded if: • Additionally, diagnosed with other DM type. • Insufficient material on DBS card • Twin, triplet etc.	A) Case-Cohort study: • Born 1981-2002 • Free of DT1 up to the end of the follow up period (2012) • Randomly selected data and biobank material from Danish Childhood Diabetes Registry B) Case-Control study: • Born 1981-1999 • DBS card next to the “case’s” card in the DNSB, matching on date and season. • “Case-control” matching was 1:1
**Talaat et al. (2016)**	• DT1 diagnosis • Afebrile for 2 weeks • Free of infectious diseases • Free of autoimmune disorders • Free of asthma, eczema, and allergies	• DT1 and DT2free • No relatives with T1D, T2D or other autoimmune disorder • “Cases-controls” were matched by sex and age • “Case-control” matching was 1:1
**Cadario et al. (2015)**	• DT1onset up to 10 years old from 2000-2012 • Blood sample collected the 3^rd^ day of birth	• Nondiabetic • “Case-control” matching was 1:5 • Matching based on date of birth (±30 days), birthplace, nationality,and parent birthplace.
**Franchi et al. (2013)**	• Newly DT1 diagnosed between 5/2010-7/2012 T1D diagnosis criteria: • SD symptoms • Glu ≥11.1 mmol/lt or fasting glu ≥7.0 mmol/lt or 2h postloadglu ≥11.1 mmol/lt • DT1diagnosisbyphysician • If no “control” matching, the “case” was excluded	• Children that visited the hospital for other health issues • Matching was based on sex, age, BMI, ethnicity, and birth season. • “Case-control” matching was 1:3
**Bener et al. (2009)**	• Glu ≥ 6.7 mmol/ltor insulin dependent • Age<16 years	• Glu <6.1 mmol/lt and never taking diabetic medication. • Visited the hospital for other health issues than acute or chronic disease. • Matching was based on sex, age, and ethnicity

In some studies, the results for 25(OH)D levels are expressed in ng/ml, whereas in others they are expressed as nmol/lt. For better interpretation and comparison of results, all 25(OH)D levels have been converted to nmol/lt, with the ratio: 1 nmol/lt corresponds to 2,496 ng/ml [27]. Four studies defined a vitamin D deficiency, 25(OH)D levels <50 nmol/lt [22-24, 27], one study <75 nmol/lt [28], and one study <25 nmol/lt [25]. Three studies defined vitamin D insufficiency 25(OH) D levels between 50 and 75 nmol/lt [22,24,27] and 4 studies defined vitamin D normal (sufficient) 25(OH)D levels >75 nmol/lt [22,24,27,28]. Five studies compared means of 25(OH)D levels [22-24,26,28] and two studies compared the medians [25,27]. The 25(OH)D levels in five studies were found to be lower in the cases group compared to the controls [22-24,27,28]; the other two studies found no statistically significant differences between the groups [25,26]. Odds ratios were used as a common measure of association in all included studies.

### 3.2 25(OH)D levels and diabetes mellitus Type 1

Overall, five studies reported a statistically significant association between 25(OH)D serum levels and DT1 [22-24,27,28], but two studies did not find a statistically significant association [25,26]. Children with normal 25(OH)D serum levels (>75 nmol/lt) in childhood had 32% lower odds (OR=0.68, 95% CI: 0.52-0.89) for auto-antibody appearance, and thus, development of T1D, than did children with 25(OH)D deficiency (<50 nmol/lt); children with normal 25(OH) D in infancy had 40% lower odds (OR=0.59, 95% CI: 0.44-0.79) than did children with 25(OH)D deficiency (<50 nmol/lt) [22]. Children with 25(OH)D 51-74 nmol/ lt had almost 4 times greater odds for developing DT1 (OR=3.45, 95% CI: 1.02-11.72, p=0.010); children with 25(OH)D ≤ 50 nmol/lt had nearly 6 times greater odds for developing DT1 (OR=5.56, 95% CI: 1.1-16.3, p=0.010) [27]. Children with 25(OH)D ≤30 nmol/lt had 1.4 greater odds for developing DT1 (OR=1.385, 95% CI: 1.0-1.9, p=0.048) than did children with normal 25(OH)D [28].

In a different study, children with DT1 had a significantly lower 25(OH)D than did healthy children (OR=4.18, 95% CI: 1.15-15.20, p=0.027) [24], whereas another study indicated that healthy children had a 50% higher 25(OH)D than did children with DT1 (OR=0.51, 95% CI: 0.33-0.68, p=0.001) [23]. In addition, one study found that girls with DT1 had significantly lower 25(OH) D (OR=0.50, 95% CI: 0.30-0.83, p=0.008) than boys [23]. Lastly, in one study, 26% of the participants with DT1 were immigrants from North African countries. In this group of migrants, researchers found that children with 25(OH)D <5.35 nmol/lt had14 times greater odds for developing DT1 until the age of 10 (OR=14.02, 95% CI: 1.76-111.7, p=0.04), than the children of the same group with 25(OH)D >5.35 nmol/lt [26].

### 3.3 Methodological quality

The methodological quality of the studies included in the systematic review was evaluated using the NOS [21]. Quality score points applied for each of nine different measures under broader category headings of participant selection, comparability between cases and controls, and their exposure (ascertainment and non-response rate). Meeting all criteria in a category would confer a maximum score of 9 stars. The maximum quality score was 9, and the range of scores was from 7 to 9. Almost all studies included were scored as high quality. All studies reported adequate case and control definition, comparability of cases and controls, and exposure ascertainment; however, most of the studies did not report their non-response rate, and subsequently, lost a score point. Table 3 presents the quality assessment scores of the included studies.

**Table 3.** Quality evaluation of the studies included in the systematic review using Newcastle Ottawa quality scale Author, year

	Selection			Comparability			Exposure		
Author, year	(1) Is the case definition adequate?	(2) Representativeness of the cases	(3) Selection of Controls	(4) Definition of Controls	(1) Comparability of cases and controls on the basis of the design or analysis ^a^	(1) Ascertainment of exposure	(2) Same method of ascertainment for cases and controls	(3) Non Response rate	Total quality score
Norris et al (2018)	⊠	⊠	⊠	⊠	⊠⊠	⊠	⊠		8
Rasoul et al (2016)	⊠	⊠	⊠	⊠	⊠⊠	⊠	⊠		8
Jacobsen et al (2016)	⊠	⊠	⊠	⊠	⊠	⊠	⊠	⊠	8
Taalat et al (2016)	⊠	⊠	⊠	⊠	⊠	⊠	⊠		7
Cadario et al (2015)	⊠	⊠	⊠	⊠	⊠⊠	⊠	⊠	⊠	9
Franchi et al (2014)	⊠	⊠	⊠	⊠	⊠⊠	⊠	⊠		8
Bener et al (2009)	⊠	⊠	⊠	⊠	⊠⊠	⊠	⊠		8

a A maximum of 2 stars can be awarded for this item. A study controlling for age receives one star, and a study controlling for other major risk factors receives an additional star.

## 4 Discussion

This systematic review assesses vitamin D deficiency as a possible cause of type 1 diabetes in DT1 in children and adolescents aged 0-15. Most studies in this systematic review reported a statistically significant association between 25(OH)D serum levels and DT1 [22-24,27,28]; only two studies did not find statistically significant differences [25,26]. Also, most studies showed lower serum levels of 25(OH)D in children with DT1 compared to those in the control group [22-24,27,28]; some of the studies we reviewed reported that children with DT1 had a higher frequency of vitamin D deficiency than did ones in the controls [22,24,25]. Similar conclusions also have been reported in other studies that we did not consider for inclusion in this systematic review as they did not provide the required measures of association (e.g., OR, RR, etc.) [29-37].

All studies included assessed 25(OH)D serum levels, which are considered the best indicator of vitamin D levels in humans and have a half-life of approximately 3 weeks [38]. Four studies we included showed a higher incidence of vitamin D deficiency in children with DT1 than the control group [23,24,27], in which vitamin D deficiency was defined as a 25(OH)D level <50 nmol/ lt, and in one study, where vitamin D deficiency was defined as a 25(OH)D level <75 nmol/lt [28]. On the other hand, two studies [25,26] that did not find statistically significant differences between the two groups, defined vitamin D deficient 25(OH)D levels as <25 nmol/lt [25] and <35 nmol/lt [26] respectively. This deviation probably affects the results, as the higher limits increase the frequency of vitamin D deficiency and reduce the differences between the two groups. Additionally, Rasoul et al. [24] and Franchi et al. [27] measured serum 25(OH)D levels in newly diagnosed patients. There is evidence to suggest that low serum 25(OH)D levels associated with diabetic ketoacidosis (DKA) at onset of disease recover to some extent with resolution of the acidosis [39].

There is evidence that the frequent occurrence of vitamin D deficiency in patients with DT1 may be due to physiological and environmental changes as this disease affects the metabolism of vitamin D [40]. The presence of vitamin D receptor (VDR) in most immune cells (lymphocytes, monocytes, macrophages, dendritic) and activated T cells leads to the evaluation of the immunomodulatory effect of calcitriol (the biologically active form of vitamin D_3_). Calcitriol inhibits the differentiation and maturation of dendritic cells while promoting their apoptosis so that they cannot act as APCs [19]. On the other hand, calcitriol stimulates the differentiation and activation of macrophages, thereby promoting their antimicrobial activity. It has been reported that calcitriol regulates the production of cytokines. Subtype 1 T-helper cell cytokines (Th1) are known to be related to cell immunity and subtype 2 T-helper cell cytokines (Th2) are related to humoral immune response [41]. Calcitriol increases the production of anti-inflammatory cytokines (e.g., interleukin 4 (IL-4), interferon 10 (IL-10), IL-1, IL-6, IL-11, and IL-13 [17,42,43], and reduces the production of Th1 cells, which are responsible for the death of beta-cells [44].

Two studies of this systematic review did not find a statistically significant association between vitamin D levels and the development of DT1 up to age 10 [26] or up to age 18 [25], respectively. This finding may be because vitamin D levels have been assessed only at birth. During childhood or adolescence, or even in adulthood, vitamin D levels may have a different role in the development of DT1 than at birth. Studies measuring 25(OH)D levels during pregnancy or evaluating the consumption of vitamin D-enriched foods during pregnancy and the risk of developing DT1 have not been concluded. Sorensen et al. reported significantly low 25(OH)D levels of Norwegian pregnant women whose infants developed DT1, compared to those women whose infants did not develop DT1 [45]. Another study reported that children (boys, but not girls) whose mothers did not consume enriched margarine during pregnancy had a 1.5-2 times higher risk of developing DT1 by the age of 14, than children whose mothers consumed enriched margarine [46]. A similar study did not demonstrate statistically significant differences between vitamin D levels during pregnancy between mothers whose children developed DT1 by the age of 7 compared to healthy children [47]. Similarly, another study had found no statistically significant association between vitamin D levels or intake of cod liver oil during pregnancy and the risk of developing DT1 [48]. Accordingly, in the study by Cadario et al. included in this systematic review, they reported that 25(OH)D levels at birth were not related to the development of DT1 by the age of 10, although in this study, levels 25(OH)D at birth varied significantly between DT1 children and children in the control group [26]. It is important to note that for Cadario et al. [26], as well as for Jacobsen et al. [25], vitamin D status was derived from neonatal dried blood spots, and the subjects followed over time to assess any association with 25(OH)D levels and development of type 1 diabetes. Neonatal blood spots would be closely associated with maternal vitamin D status and would not necessarily be predictive of vitamin D status in early childhood, childhood, or adolescence. This may be why the authors of these two studies found no association between neonatal vitamin D status and subsequent diabetes risk.

The use of vitamin D supplements in infancy seems to have a greater effect on the risk of developing DT1 than exposure to vitamin D during pregnancy. A meta-analysis showed that the use of vitamin D supplements in infancy reduces the risk of developing DT1 in later life [49]. The timing of regulating vitamin D levels is crucial for achieving the maximum result. Infants aged 7 and 12 months who received a vitamin D supplement had almost half the risk of developing DT1 than infants who received the vitamin supplement up to the age of 6 months [50]. This finding may be because the adaptive immunity mechanisms become active during the second half of the first year of life. In the first 6 months of life, therefore, vitamin D probably has no regulatory action [51].

Many interventional studies and randomized controlled trials show positive results in patients with DT1 when insulin intake is combined with vitamin D supplementation (D_3_), as there is an improvement in beta-cell function [52-54]. Vitamin D_3_ supplementation has been shown to improve glycemic control in patients with DT1, significantly reducing HbA1c after 3 months of treatment in different dosages [55-57]. D_3_ supplementation has a protective effect both in patients with a recent DT1 diagnosis and in patients with a diagnosis of longer duration. An increase in T regulatory cells was observed in patients who received daily 2000 IU D_3_ for one year [58] or 4000 IU for 3 months [57]. Besides, after a 12-month daily intake of 70 IU per body weight kg (70 IU/kg), an increase was observed in the sedative ability of T regulator cells in the treated subjects than those who received placebo [59]. An improvement in the glycemic profile of children with DT1 was observed after a three-month intake of 4000-10,000 IU/day [52, 56] or 50,000 IU/week [55]. No statistically significant effect of vitamin D supplement intake was found in two randomized double-blind clinical trials between patients with a recent DT1 diagnosis and a control group. In particular, the daily supplementation of 0.25μg of calcitriol for 18-24 months was not found to have an effect on the function of beta-cells [60, 61]. In another interventional study, a single dose of 100,000 IU or 160,000 IU of D_3_ did not appear to influence glycemic control of patients with DT1 [62].

### 4.1 Strength and limitations

The main strength of our study is that it is the first systematic review, to our knowledge, to evaluate the role of vitamin D levels in the development of DT1 in children and adolescents aged 0-15. Another strength of this systematic review is that it has been conducted in a transparently; the literature search and selection was based on specific criteria and the articles included had a high-quality score according to the NOS. Nevertheless, some limitations need to be considered. First, it is difficult to ensure that all the relevant literature has been identified. Nevertheless, to avoid missing important studies, the reference lists of relevant studies were screened to identify additional studies. Second, we were unable to perform a quantitative meta-analysis due to substantial between-study heterogeneity in study methodology (e.g., measurement methods, exposure definitions, follow-up duration). Vitamin D deficiency and insufficiency were defined in different levels in most studies, and this could affect the results, as higher limits increase the frequency of vitamin D deficiency and reduce the differences between the two groups (patients and controls). This limitation could be minimized with the use of standardized definitions and protocols for vitamin D levels (normal, insufficiency, deficiency). Another limitation included the variation between the follow-up periods. The follow-up periods ranged from 6 months to 30 years and the number of participants ranged from 58 to 2873, leading to difficulty in drawing conclusions. In addition, bias among studies may have occurred because they were carried out in different countries and seasons of the year, as ultraviolent B is the main source of vitamin D for the human body, and sunlight differs by country and by season of the year. Lastly, the non-response rate was not reported in most studies, indicating a high risk for attrition bias.

### 4.2 Conclusion

In conclusion, this systematic review demonstrates that vitamin D deficiency could be a possible cause of DT1 in early life, particularly in children with a genetic predisposition. Moreover, we found that vitamin D deficiency is a common occurrence in patients with DT1, whereas normal vitamin D levels seem to reduce the risk of DT1 in later life. The observational nature of the included studies, which are often more susceptible to different types of bias as well as the substantial between-study heterogeneity, may reduce the reliability of our conclusions. This highlights the need for standardized definitions in exposures and outcomes as well as the adoption of reporting guidelines, e.g., Strengthening the Reporting of Observational Studies in Epidemiology (STROBE) [63] in future studies to improve the research evidence in this field. Furthermore, long-term follow-up studies with large sample sizes are required to assess the role of vitamin D more effectively as a possible factor in the development of DT1.

## Data Availability

The dataset used and analysed during the current study is available from the corresponding author on reasonable request.

## Abbreviations

APCs: Antigenic precenting cells

CDC: Centre for disease prevention and control

DT1: Diabetes mellitus type 1

DM: Diabetes mellitus

HR: Hazard Ratio

IL-4: Interleukin 4

IL-10: Interferon 10

NOS: Newcastle-Ottawa scale

OR: Odds Ratio

PRISMA: Preferred reporting items for systematic reviews and meta-analyses guidelines

RR: Risk ratio

Th1: 1 T-helper cell cytokines

Th2: 2 T-helper cell cytokines

TNF-a: Necrosis factor-alpha

USA: Unites states of America

UVB: Ultraviolet sunlight

VDR: Vitamin D receptor
